# Myc activity is required for maintenance of the neuromesodermal progenitor signalling network and for segmentation clock gene oscillations in mouse

**DOI:** 10.1242/dev.161091

**Published:** 2018-07-30

**Authors:** Ioanna Mastromina, Laure Verrier, Joana Clara Silva, Kate G. Storey, J. Kim Dale

**Affiliations:** Division of Cell and Developmental Biology, School of Life Sciences, University of Dundee, Dow Street, Dundee DD1 5EH, UK

**Keywords:** Myc, Neuromesodermal progenitors, Segmentation clock, Embryo, Presomitic mesoderm

## Abstract

The Myc transcriptional regulators are implicated in a range of cellular functions, including proliferation, cell cycle progression, metabolism and pluripotency maintenance. Here, we investigated the expression, regulation and function of the Myc family during mouse embryonic axis elongation and segmentation. Expression of both *cMyc* (*Myc* – Mouse Genome Informatics) and *MycN* in the domains in which neuromesodermal progenitors (NMPs) and underlying caudal pre-somitic mesoderm (cPSM) cells reside is coincident with WNT and FGF signals, factors known to maintain progenitors in an undifferentiated state. Pharmacological inhibition of Myc activity downregulates expression of WNT/FGF components. In turn, we find that *cMyc* expression is WNT, FGF and Notch protein regulated, placing it centrally in the signalling circuit that operates in the tail end that both sustains progenitors and drives maturation of the PSM into somites. Interfering with Myc function in the PSM, where it displays oscillatory expression, delays the timing of segmentation clock oscillations and thus of somite formation. In summary, we identify Myc as a component that links NMP maintenance and PSM maturation during the body axis elongation stages of mouse embryogenesis.

## Abstract (precis)

**Highlighted Article:** Myc operates in a positive feedback loop with WNT/FGF signals to maintain the progenitors which facilitate body axis elongation, and its activity is crucial for timing of the segmentation clock.

## INTRODUCTION

The Myc proto-oncogene family is one of the most exhaustively studied families of vertebrate genes (Eilers and Eisenman, 2008; Meyer and Penn, 2008). Since the discovery of *cMyc* (in chick) (Alitalo et al., 1983; Watson et al., 1983), two more members were identified, namely *MYCN* (Brodeur et al., 1984; Emanuel et al., 1985) and *L-MYC* (*MYCL* – Human Gene Nomenclature Database) (Ikegaki et al., 1989; Nau et al., 1985), and a plethora of studies has placed each member centrally in tumorigenesis, in a context-specific manner (Tansey, 2014). It is now established that the oncogenic potential of Myc is mediated through the transcriptional control of multiple target gene sets (Dang et al., 2006; Zeller et al., 2003, 2006). Myc contains a basic helix-loop-helix (bHLH) domain and transcriptional activation takes place when it heterodimerizes with Max (Blackwood and Eisenman, 1991; Blackwood et al., 1991), and repression when it dimerizes with Miz1 (Staller et al., 2001). Additional co-factors, such as the bromodomain-containing protein BRD4, mediate recruitment of the Myc complex onto the chromatin (Delmore et al., 2011).

The discovery of *cMyc* as one of the four Yamanaka factors (Takahashi and Yamanaka, 2006) has highlighted multiple roles for Myc within the pluripotent cell state (Fagnocchi and Zippo, 2017). During embryogenesis, Myc has been implicated in the metabolic regulation of the pre-implantation embryo (Scognamiglio et al., 2016), progenitor sorting and cell competition in the early postimplantation epiblast (Clavería et al., 2013; Sancho et al., 2013), maintenance of the neural crest progenitor pool (Kerosuo and Bronner, 2016) and neural differentiation progression (Zinin et al., 2014).

Both *cMyc* and *MycN* homozygote mutant mice are embryonic lethal, displaying a range of defects (Davis et al., 1993; Sawai et al., 1993; Trumpp et al., 2001), suggesting that the Myc factors hold important roles during development and, likely, in a context-specific manner. Expression pattern analyses indicate the presence of both *cMyc* and *MycN* in multiple embryonic tissues (Downs et al., 1989; Kato et al., 1991; Ma et al., 2014). However, these data, based on radiolabelled probes, give very low definition and low signal-to-noise ratio, and, as such, cannot be utilized to decipher precise patterns of expression. For example, detailed expression pattern and specific functions of the Myc genes during elongation and segmentation of the embryo body axis has yet to be investigated, with respect to the different progenitor subpopulations that comprise the tail region (Wymeersch et al., 2016). In particular, the embryonic day (E) 8.5 postimplantation epiblast is a heterogeneous domain in which progenitors with different developmental potentials reside (Henrique et al., 2015; Wilson et al., 2009; Wymeersch et al., 2016).

Key to this study, detailed fate mapping and clonal analysis has indicated that posterior neural and mesoderm lineages emerge from a common progenitor population, termed the neuromesodermal progenitors (NMPs) (Cambray and Wilson, 2002; Cambray and Wilson, 2007; Delfino-Machín et al., 2005; Tzouanacou et al., 2009). NMPs have been identified in human, mouse, chicken and zebrafish embryos (Goto et al., 2017; Olivera-Martinez et al., 2012; Wymeersch et al., 2016), and have been generated *in vitro* from both mouse and human embryonic stem cells (ESCs) (Gouti et al., 2017; Gouti et al., 2014; Tsakiridis et al., 2014; Turner et al., 2014; Verrier et al., 2018). In the mouse embryo, NMPs first arise at E7.5, in the domain of the node streak border (NSB) and associated caudal-lateral epiblast (CLE), persist in the NSB and CLE at E8.5, and are subsequently incorporated in the chordo-neural hinge (CNH) during tail growth stages (Henrique et al., 2015). Importantly, the dual-fated NMPs supply cells to both the forming neural plate (open pre-neural tube) and to the caudal pre-somitic mesoderm (cPSM) (Gouti et al., 2014; Rodrigo Albors et al., 2016 preprint; Tzouanacou et al., 2009), which further matures and segments rostrally to form the somites. The NMPs and cPSM cells are maintained in an ‘undifferentiated’ progenitor state, mainly through the activity of WNT and FGF signals, components of which show very high expression in the posterior of the embryo (Hubaud and Pourquié, 2014; Wilson et al., 2009). In addition, WNT, FGF and Notch signalling pathways comprise the segmentation clock, a molecular oscillator which regulates the periodic segmentation of the pre-somitic mesoderm (PSM) into somites (Hubaud and Pourquié, 2014; Maroto et al., 2012). Concomitantly, neural and somitic differentiation is promoted by retinoic acid (RA), which is produced by the somatic tissue and counteracts WNT/FGF signalling (Delfino-Machín et al., 2005; Dequeant and Pourquie, 2008; Diez del Corral et al., 2003; Dubrulle and Pourquié, 2004; Naiche et al., 2011; Olivera-Martinez and Storey, 2007; Sakai et al., 2001). Interestingly, *cMyc* has been shown to be present and to display dynamic oscillatory mRNA expression in the PSM (Dequeant et al., 2006; Krol et al., 2011), while also being expressed at high levels in the domain that harbours the NMPs in the chicken embryo (Olivera-Martinez et al., 2014). However, no investigation as to the functional significance of Myc expression in these domains has been conducted.

Here, we elucidate divergent roles for Myc during posterior embryonic body axis formation. We find that cMyc is indispensable for the proper timing of clock gene oscillations through regulation of Notch signalling. Moreover, we demonstrate that Myc operates in a positive feedback loop with WNT and FGF signalling in the CLE of the E8.5 embryo, and that inhibition of Myc activity results in transcriptional downregulation of different gene sets, which include regulators of metabolism. These findings are the first to provide a common regulator of different sets of genes that coordinate progenitor cell maintenance, metabolism and differentiation in the NMPs and cPSM in the mouse embryo.

## RESULTS

### *cMyc* is expressed in the CLE and underlying cPSM and its expression persists during axial elongation and body axis segmentation

**Fig. 1.**
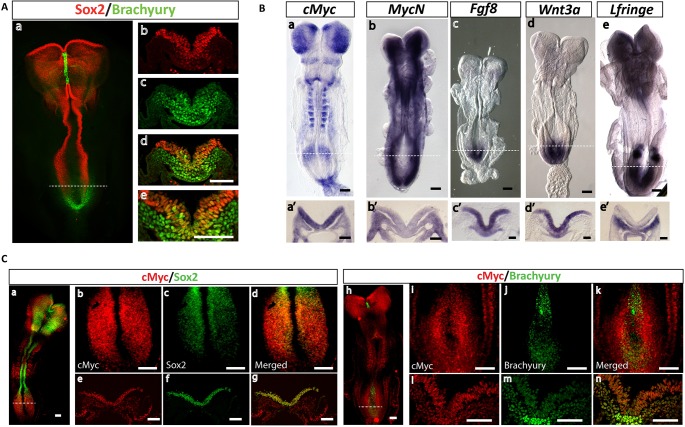
***cMyc* is co-expressed with *Wnt3a*, *Fgf8*, *Sox2* and brachyury in the CLE.** (A) Representative confocal images of an E8.5 embryo labelled by immunofluorescence for Sox2 and brachyury (*n*=3 embryos). (a) Whole-mount E8.5 embryo and (b-d) transverse sections at the level of the CLE domain (demarcated by the white dashed line in a). Sox2 labels the neuroepithelium along the anterior-posterior axis and the CLE, whereas brachyury labels the PSM and tail bud mesoderm. (e) Magnification of d, showing the location of the NMPs (Sox2/brachyury co-expressing cells) in the CLE epithelium. (B) Representative *in situ* hybridization (ISH) images of E8.5 embryos labelled for (a) *cMyc* (*n*=10 embryos), (b) *MycN* (*n*=4 embryos), (c) *Fgf8* (*n*=3 embryos), (d) *Wnt3a* (*n*=3 embryos) and (e) *Lfringe* (*n*=5 embryos). (a′-e′) Transverse sections of the CLE and underlying cPSM domain indicated by the white dashed lines in a-e. (a′) *cMyc*, (c′) *Fgf8* and (d′) *Wnt3a* show high levels of expression in the CLE. (b′) *MycN* and (e′) *Lfringe* show high levels of expression in the cPSM. (C) Representative confocal images of immunofluorescence labelling of E8.5 embryos for cMyc and Sox2 (a-g; *n*=3 embryos) and cMyc and brachyury (h-n; *n*=3 embryos). Sox2/cMyc co-expressing cells are evident in the transverse sections of the CLE (e-g; panels correspond to sections at the level of the domain demarcated by the white dashed line in a). Brachyury/cMyc co-expressing cells are evident both in the CLE and underlying cPSM (l-n; panels correspond to the white dashed line in e). Scale bars: 100 μm.

We generated *cMyc* and *MycN* riboprobes and carried out an initial expression pattern analysis (Fig. 1). We were particularly interested to see that both Myc members show high levels of expression in the posterior of the embryo proper. We find high levels of *cMyc* in the CLE domain, and lower levels in the underlying cPSM (Fig. 1Ba,a′). *MycN* exhibits a complementary expression pattern: low in CLE and higher in the underlying cPSM (Fig. 1Bb,b′). The CLE is the region in which a small bipotent population of precursors is located, namely the NMPs. These cells can be visualized by the co-expression of Sox2 and brachyury (Fig. 1A) and maintenance of their bipotency relies on autocrine and paracrine WNT/FGF signalling. We find that both Myc factors are expressed alongside *Wnt3a* and *Fgf8* in the CLE and alongside the Notch target gene *Lfringe* (*Lfng*) (Dale et al., 2003; McGrew et al., 1998) in the cPSM (Fig. 1B)*.* Using immunofluorescence, we find that cMyc is co-expressed with Sox2 and brachyury in the CLE and underlying cPSM (Fig. 1C). These expression data therefore show that mouse NMPs co-express cMyc, WNT3A, FGF8, Sox2 and brachyury. Crosstalk between cMyc and FGF (Yu et al., 2017) or WNT (Fagnocchi et al., 2016) proteins or Sox2 (Lin et al., 2009) has been reported in other systems. It is therefore likely that cMyc might be involved in the NMP signalling network.

### *cMyc* is expressed in the tail bud at E9.5 and E10.5 and displays oscillatory mRNA expression in the PSM

**Fig. 2.**
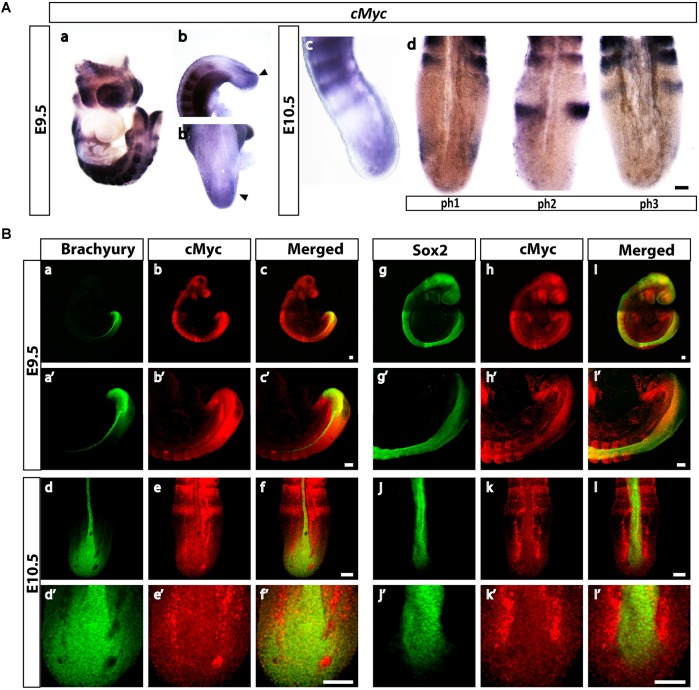
**cMyc expression persists in the tail bud during E9.5-E10.5 and shows dynamic expression at the transcript level.** (A) Representative ISH images of a tail bad at E9.5 and E10.5. (a) Whole-mount E9.5 embryo labelled for *cMyc* at E9.5 (*n*=12 embryos). (b,b′) High levels of cMyc are present in the caudal-most neuroepithelium and adjacent PSM (arrowheads). (c) Side view of an E10.5 tail labelled for *cMyc* mRNA. (d) Three different expression profiles for *cMyc* in the PSM of E10.5 embryos, reminiscent of the three phases of segmentation clock gene expression (*n*=10 embryos). (B) Representative confocal images of immunofluorescence labelling for cMyc in E9.5 and E10.5 embryos. (a-f) cMyc and brachyury staining in whole-mount embryos. (a′-f′) Higher magnification images of a-f showing cMyc/brachyury co-expressing cells in the tail bud (*n*=5 embryos). (g-l) cMyc and Sox2 labelling in whole-mount embryos. (g′-l′) Higher magnification images of g-l, showing cMyc/Sox2 co-expressing cells in the tail bud (*n*=5 embryos). Scale bars: 100 μm.

We further characterized expression of *cMyc* and *MycN* during E9.5 and E10.5, the embryonic stages in which the anterio-posterior axis elongates and segments into somites (Gibb et al., 2010; Henrique et al., 2015). The tail bud mesoderm is the main reservoir of cPSM progenitors, whereas the caudal-most, Sox2/brachyury-positive region of the neuroepithelium harbours the NMPs. Using *in situ* hybridization (ISH), we find that *cMyc* displays dynamic mRNA expression in the PSM, reminiscent of clock gene expression (Fig. 2Ad), consistent with previously published data in mouse and chick PSM (Dequeant et al., 2006; Krol et al., 2011). We find that cMyc protein is expressed in the caudal-most neuroepithelium (labelled by Sox2 and brachyury) and adjacent tail bud mesoderm (labelled by brachyury) (Fig. 2B). *MycN* is expressed in the E9.5 tail bud; however, its expression is downregulated at E10.5 and E11.5 (Fig. S1).

### Suppression of Myc activity attenuates expression of key FGF/WNT network components, leading to loss of NMP identity

**Fig. 3.**
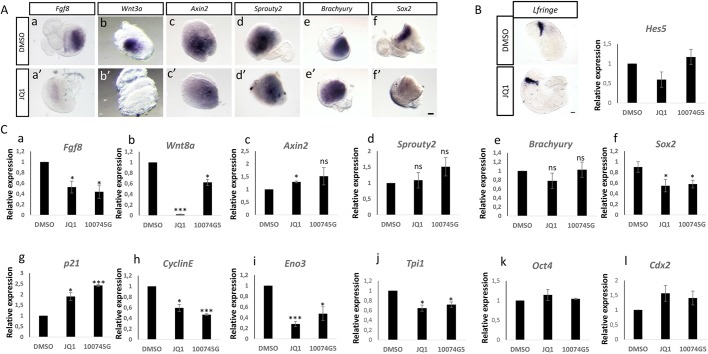
**Myc activity suppression results in downregulation of *Wnt3a/8a*, *Fgf8* and *Sox2*.** (A) Representative ISH images of CLE/cPSM explants treated with DMSO (a-f) or 10 μM JQ1 (a′-f′) for 6 h. *Fgf8* (a,a′; *n*=8/8 embryos), *Wnt3a* (b,b′; 5/5 embryos) and *Sox2* (f,f′; 3/3 embryos) expression is suppressed upon JQ1 treatment. In contrast, expression of *Axin2* (c,c′; *n*=4/4 embryos), *Sprouty2* (d,d′; *n*=4/4 embryos) and brachyury (e,e′; *n*=6/6 embryos) is not affected. (B) Representative ISH images of half-tail explants from E8.5 embryos (micro-dissected below the level of the last somite pair), treated either with DMSO or 10 μΜ JQ1 for 6 h, showing no effect on expression of the Notch target gene *Lfringe* (*n*=4/4 embryos). RT-qPCR analysis of CLE/cPSM explants for *Hes5* expression show no differences upon treatment with 10 μΜ JQ1 or 75 μΜ 10074G5 for 6 h. (C) Characterization of gene expression changes in CLE/cPSM explants upon 10 μΜ JQ1 or 75 μΜ 10074G5 for 6 h. Relative gene expression, normalized to actin levels, is shown. Data are from three independent experiments, presented as mean±s.e.m. Statistical significance was assessed using the unpaired two-tailed Student’s *t*-test for samples with unequal variance. Scale bars: 100 μm.

A small molecule approach was used to investigate whether Myc activity regulates expression of key components of the WNT/FGF/Notch network in the CLE/cPSM. To this end, we micro-dissected explant pairs that contained the NMPs and underlying cPSM from E8.5 embryos, and cultured them for 6 h in the presence of small molecule inhibitors that have been extensively used to interfere with Myc function *in vitro* (Delmore et al., 2011; Horne et al., 2014; Posternak and Cole, 2016; Yin et al., 2003). Two different small molecules, which act via distinct molecular mechanisms, were used to cross-validate the specificity of our findings: JQ1 is a small molecule that competitively binds to BRD4, a co-factor that recruits the Myc complex onto the chromatin (Delmore et al., 2011); 10074G5 interferes with heterodimerization of Myc with its binding partner, Max (Yin et al., 2003). As a readout of inhibitor efficacy, we quantified – by quantitative real-time polymerase chain reaction (RT-qPCR) – the expression levels of two well-established Myc targets, cyclin E1 and *p21* (*Cdkn1a*) (Zeller et al., 2003), and found that upon treatment with either inhibitor, *p21* levels were significantly increased, whereas cyclin E1 levels significantly decreased (Fig. 3g,h). This is consistent with negative and positive regulation of *p21* and cyclin E1 expression, respectively, as reported previously (Claassen and Hann, 2000; Gartel et al., 2001; Pérez-Roger et al., 1997).

We then assessed expression levels of key WNT, FGF and Notch pathway components using RT-qPCR and ISH. Following Myc inhibition, a sharp downregulation of *Fgf8*, *Wnt3a/8a* and *Sox2* transcripts was observed (Fig. 3Aa-b′,f,f′,Ca,b,f). Importantly, *Axin2*, *Sprouty2* (*Spry2*), *Lfringe* and *Hes5* expression levels were unaltered at this 6 h timepoint, revealing that despite the reduction in FGF and WNT ligand transcripts, WNT, FGF and Notch target gene expression is not compromised (Fig. 3Ac-d′,B,Cc,d). In addition, even though *Sox2* expression (indicative of NMP identity in this domain) is affected in the explants, the core epiblast identity [as judged by *Cdx2* and *Oct4* (*Pou5f1*) mRNA expression; Deschamps and Duboule, 2017] is not affected (Fig. 3Ck,l). Additionally, we quantified the expression levels of several metabolic genes identified recently to show high expression in the tail bud (Oginuma et al., 2017), and found that two of them, triosephosphate isomerase 1 (*Tpi1*) and enolase 3 (*Eno3*), show significant downregulation upon Myc activity suppression, consistent with Myc controlling the expression of glycolytic genes in other contexts (Hsieh et al., 2015; Kim et al., 2004; Stine et al., 2015) (Fig. 3Ci,j).

**Fig. 4.**
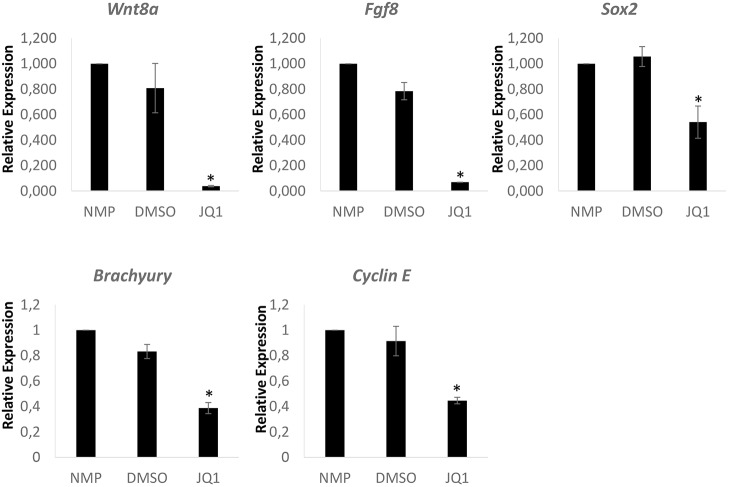
**MYC activity suppression in *in vitro*** **human NMPs** **results in downregulation of *FGF8*,** ***WNT8A*,** ***SOX2* and** **brachyury.** 500 nM of JQ1 was applied for 24 h. Relative gene expression, normalized to expression of *PRT2* is shown. Data are from three independent experiments, presented as mean±s.e.m. Statistical significance was assessed using the unpaired two-tailed Student’s *t*-test for samples with unequal variance. *P<0.05.

To further corroborate our hypothesis that Myc is important for the maintenance of WNT/FGF signalling we repeated this investigation in an NMP-like cell population generated *in vitro* from human ESCs (hESCs) (Verrier et al., 2018). Using this protocol, SOX2/brachyury co-expressing cells can be generated with high efficiency, while extensive gene expression characterization, including RNA sequencing, indicates that these cells faithfully represent the embryonic NMPs (Verrier et al., 2018). Successful differentiation to the NMP state was verified by immunofluorescence showing co-expression of Sox2 and brachyury, and co-expressing cells could be maintained *in vitro* for 24 h (Fig. S3). Treatment with 500 nM JQ1 for 24 h resulted in significant downregulation of *SOX2*, brachyury, *WNT8A*, *FGF8* and cyclin E1, despite the excess of WNT/FGF proteins that are present in the culture medium of the human NMP-like cells (Fig. 4).

Taken together, these data indicate a specific requirement for MYC-dependent transcription of key NMP maintenance factors, namely *WNT3A/8A*, *FGF8* and *SOX2*.

### Alleviation of Myc inhibition is required for neural and mesodermal differentiation

**Fig. 5.**
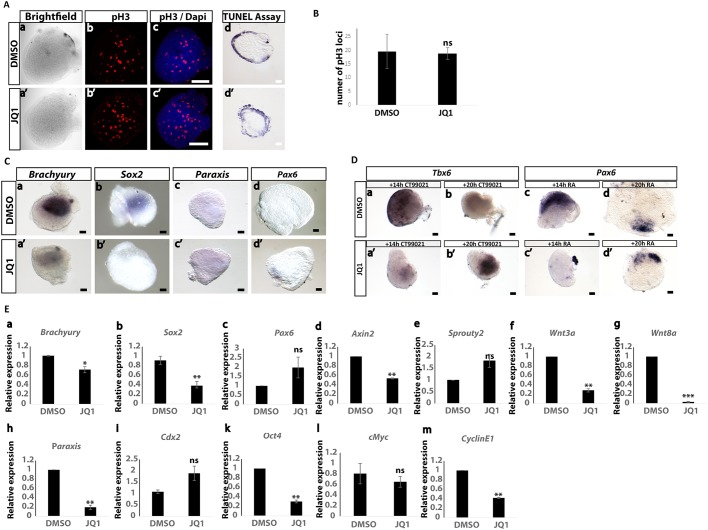
**Myc activity suppression does** **not promote differentiation, nor does it compromise viability and proliferation.** (A) Representative confocal images of CLE/cPSM explants cultured for 10 h, either in DMSO or in 10 μΜ JQ1 and subsequently stained for pH3 by immunofluorescence (*n*=3 embryos). The TUNEL assay was employed to analyse cell death. Apoptotic nuclei were detected in peripheral edges of both DMSO- and JQ1-treated explants (*n*=3 embryos). (B) No significant differences in the number of pH3-positive loci were found between DMSO- and JQ1-treated explants (explants micro-dissected from three different embryos, quantified in 18 optical sections per condition). Data are presented as mean±s.e.m. (C) CLE/cPSM explants treated in DMSO (a-d) or with 10 μΜ JQ1 (a′-d′) for 10 h analysed by ISH. At this timeframe, both *Sox2* (*n*=3/3) and brachyury (*n*=7/10) expression is downregulated, and differentiation markers such as *Pax6* (*n*=0/5 embryos) and *Paraxis* (*n*=0/3 embryos) are not expressed in control DMSO and JQ1-treated explants. (D) CLE/cPSM explants after 10 h of culture in DMSO (a-d) or in 10 μΜ JQ1 (a′-b′). Representative ISH images of explants treated with CT99021 (a,b,a′,b′) or 100 nM RA (c,d,c′,d′) for 14 h or 20 h following the 10 h culture in DMSO or 10 μΜ JQ1. Transient Myc suppression for 10 h prior to 14 h of CT99021 stimulation (a′) causes low levels of *Tbx6* mRNA (*n*=6/6 embryos) compared with DMSO treatment alone (a′). High levels of *Tbx6* when CT9901 exposure was prolonged to 20 h (*n*=4/4 embryos) (b-b′). Explants transiently incubated with JQ1 express no *Pax6* after 14 h of RA treatment (*n*= 0/6 embryos), in contrast to the DMSO control explants. Explants transiently incubated with JQ1 do express *Pax6* after 20 h of RA treatment (*n*= 5/5 embryos), as do the control explants. (E) RT-qPCR analysis of gene expression changes in control CLE/cPSM explants cultured in DMSO or in 10 μΜ JQ1 for 10 h. Relative gene expression, normalized to actin levels, is shown. Data are from three independent experiments, presented as mean±s.e.m. Statistical significance was assessed using the unpaired two-tailed Student’s *t*-test for samples with unequal variance. *P<0.05, **P<0.01, ***P<0.001; ns, nonsignificant. Scale bars: 100 μm.

We next investigated the possibility that transcriptional downregulation of WNT and FGF protein ligands, following Myc inhibition, promotes precocious differentiation. Therefore, culture with 10 μΜ JQ1 was increased to 10 h. Neither *Pax6* (a neural progenitor marker gene) (Stoykova et al., 1996) nor *Paraxis* (*Tcf15*) (a rostral paraxial mesoderm marker) (Burgess et al., 1995) expression was detected (Fig. 5C). This suggests either that longer culture is required for differentiation or that Myc activity is important for initiation and/or progression of differentiation. To define better the effects of Myc inhibition after 10 h we next assessed the impact of this treatment on read-outs for FGF and WNT signalling. Indeed we observed that WNT protein transduction is attenuated, as indicated by *Axin2* transcription; however, expression of the FGF target gene *Sprouty2* is not significantly affected (Fig. 5E).

The effect of Myc inhibition on cell behaviour in these assays was also addressed. Myc inhibition using JQ1 did not induce apoptosis as revealed by the terminal deoxynucleotidyl transferase dUTP nick-end labelling (TUNEL) assay, with positive cells only detected at the cut edges of explants in both treatment and control conditions (Fig. 5A). Myc orchestrates the expression of many genes involved in cell cycle progression (Dang et al., 2006; Eilers and Eisenman, 2008; Zeller et al., 2003, 2006). Analysis of the known Myc target cyclin E1 indicated a reduction in transcripts following 10 h JQ1 treatment (Fig. 5E). We therefore determined the number of phospho histone 3 (pH3)-positive cells, indicative of late G2/mitotic phase (Fig. 5A). We did not observe significant differences in this time period (Fig. 5B); however, this might be related to the cell cycle length in NMPs, estimated to be ∼7-8 h in the chicken embryo (Olivera-Martinez et al., 2014), and as such it is perhaps not surprising that we do not see proliferation impairment following 10 h of Myc activity suppression.

To determine whether the lack of precocious differentiation was due to a requirement for Myc activity for initiation/progression of differentiation, we first transiently suppressed Myc using 10 μΜ JQ1 for 10 h, as above, and subsequently washed out the inhibitor and cultured the explants for a further 14 h, either in plain culture medium or in the presence of differentiation stimuli. Washout of Myc inhibition was not sufficient to stimulate expression of differentiation markers (Fig. S2). To stimulate differentiation towards the mesoderm lineage, we employed the potent GSK3 (GSK3B) antagonist CT99021 (Cohen and Goedert, 2004). We incubated explants [previously treated for 10 h with dimethyl sulfoxide (DMSO) or JQ1] in 30 μΜ CT99021 for 14 h and then analysed expression of the cPSM marker *Tbx6* (Chapman et al., 1996). DMSO control explants showed high *Tbx6* expression, whereas JQ1-treated explants exhibited very low expression (Fig. 5Da-a′). However, prolonging exposure to CT99021 for a further 6 h (20 h in total, postremoval of Myc inhibition) induced high *Tbx6* expression in the JQ1-treated explants. In contrast, at this timepoint, the DMSO-treated explants no longer expressed *Tbx6*, likely due to their further differentiation along the paraxial mesoderm maturation pathway (Fig. 5Db-b′). We then repeated exactly the same experiment, this time stimulating retinoid signalling, to promote neural differentiation, using 100 nM RA for 14 h and 20 h as above. Similarly, JQ1-treated explants were delayed in their response to upregulate expression of the neural marker gene *Pax6* (Patel et al., 2013; Stoykova et al., 1996) in response to RA stimulation (Fig. 5Dc,c′,d,d′).

These experiments suggest that Myc directs the expression of multiple genes within the NMP/cPSM network. One of the gene sets involves core factors functioning in NMPs (*Fgf8*, *Wnt3a*, *Wnt8a*, *Sox2*) and cPSM (*Fgf8*, *Wnt3a*) progenitor pool maintenance. The other gene sets are involved in cell cycle progression (*p21*, cyclin E1) and glycolytic metabolism (*Eno3*, *Tpi1*). At the same time, Myc is required for the differentiation response to external signalling cues, likely by regulating a different target gene set.

### WNT, FGF and Notch signalling converge upstream of cMyc expression

We then explored what signals regulate cMyc expression in these domains. cMyc has been shown to be a canonical Notch and WNT protein target *in vitro* (He et al., 1998; Herranz et al., 2014; Palomero et al., 2006; Weng et al., 2006), and FGF/ERK (MAPK) genes have been proposed to act upstream of *cMyc* transcription (de Nigris et al., 2001), mRNA stability (Notari et al., 2006) and protein turnover (Kress et al., 2015; Sears, 2004). In addition, retinoid signalling has been previously shown to have a negative effect on Myc expression (Griep and DeLuca, 1986). Here, we employed gain- and loss-of-function approaches to test whether WNT, FGF or Notch proteins or RA regulate *cMyc* transcription in the CLE/cPSM domain.

**Fig. 6.**
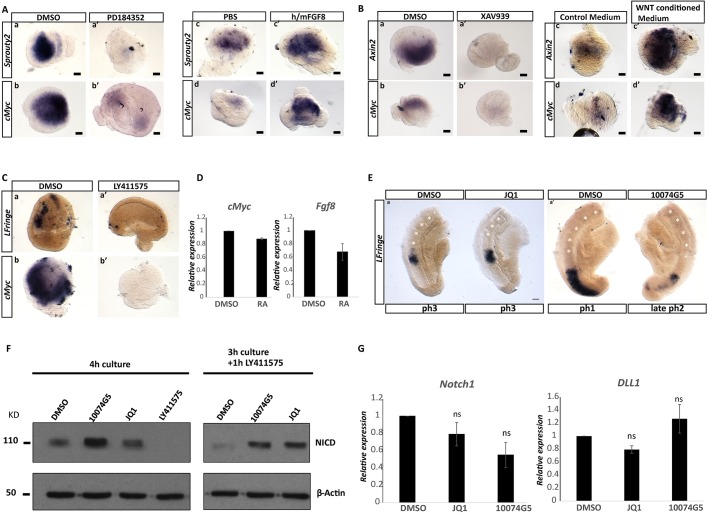
**cMyc is co-regulated by WNT, FGF and Notch signals and Myc activity suppression results in delays in somite formation.** (A) Representative ISH images of CLE/cPSM explants cultured for 4 h in DMSO (a,b) or with 3 μΜ PD184352 (a′,b′) to block ERK phosphorylation. *Sprouty2* (*n*=5/5 embryos) and *cMyc* (*n*=5/6 embryos) expression is downregulated in the PD184352-treated explants. Stimulation of FGF signalling using 250 ng/ml recombinant FGF8 protein for 8 h results in upregulation of both *Sprouty2* (*n*=5/8 embryos) and *cMyc* (*n*=5/9 embryos) in CLE/cPSM explants (c′,d′), when compared with control explants cultured in PBS (c,d). (B) Representative ISH images of CLE/cPSM explants cultured for 4 h in DMSO (a,b) or 10 μΜ XAV939 (a′,b′) to block tankyrase activity. *Axin2* (*n*=7/7 embryos) and *cMyc* (*n*=4/5 embryos) expression is downregulated in the XAV939-treated explants. Stimulation of WNT signalling using WNT3A-conditioned culture medium for 6 h results in upregulation of both *Axin2* (*n*=3/3 embryos) and *cMyc* (*n*=3/3 embryos) in CLE/cPSM explants (c′,d′) when compared with control explants cultured in DMSO (c,d). (C) Representative ISH images of E8.5 half-tail explants (micro-dissected at the level below the last formed somite) cultured for 4 h in DMSO (a,b) or CLE/cPSM explants cultured in the presence of 150 nM LY411575 (a′,b′) to block γ-secretase activity. *Lfringe* (*n*=4/4 embryos) and *cMyc* (*n*=4/4 embryos) expression is downregulated in the LY411575-treated explants compared with controls. (D) RT-qPCR quantification of *cMyc* and *Fgf8* expression in CLE/cPSM cultured for 6 h in DMSO or 100 nM RA. Relative gene expression normalized to actin levels is shown. Data are from two independent experiments, presented as mean±s.e.m. (E) Representative images of E9.5 half-tail explants treated with 10 μΜ JQ1 (a) or with 75 μΜ 10074G5 (b) for 4 h, labelled by ISH for the Notch clock gene *Lfringe*. Myc inhibition delayed *Lfringe* phase pattern expression (11/16 explants treated with JQ1, 4/5 explants treated with 10074G5). (F) Western blot analysis of NICD levels in E9.5 embryo tails. Tails treated with JQ1 or 10074G5 for 4 h show increased NICD levels when compared with control DMSO tails cultured in parallel. Culture in the presence of 150 nM LY411575 for 4 h completely abolishes NICD as expected. Addition of 150 nM LY411575 for the last 1 h of culture depletes NICD only in the DMSO sample, whereas samples treated with JQ1 or 10074G5 exhibit higher levels of NICD. (G) RT-qPCR quantification of *Notch1* and *Dll1* expression in control CLE/cPSM explants cultured for 6 h in DMSO, or in CLE/cPSM explants cultured in parallel in 10 μΜ JQ1 or 75 μΜ 10074G5, reveals no significant changes in expression. Relative gene expression, normalized to actin levels, is shown. Data are from three independent experiments, presented as mean±s.e.m. Statistical significance was assessed using the unpaired two-tailed Student’s *t*-test for samples with unequal variance. ns, nonsignificant. Scale bars: 100 μm.

We first checked whether FGF regulates cMyc by incubating CLE/cPSM explants for 4 h with 3 μΜ of the MEK inhibitor PD184352 (Allen et al., 2003) to interfere with FGF signal transduction, or with recombinant FGF8 protein to stimulate the endogenous pathway. The expression of *Sprouty2*, an FGF/ERK target gene (Sivak et al., 2005; Thisse and Thisse, 2005), was monitored in parallel as a positive control. Explants in which ERK protein activity was suppressed showed severe downregulation of both *Sprouty2* and *cMyc* expression, whereas explants exposed to exogenous FGF protein showed increased levels of both genes, indicating a role for FGF proteins upstream of *cMyc* (Fig. 6Aa-d′). Next, we tested whether WNT signalling regulates cMyc expression using 10 μΜ of the known tankyrase inhibitor XAV939 to suppress WNT signalling (Huang et al., 2009) or WNT3A-conditioned medium to stimulate WNT signalling. *Axin2* was used as a readout for WNT activity (Jho et al., 2002). Similarly to the FGF experiment, we found that *Axin2* and *cMyc* were both downregulated following XAV939 treatment and were both upregulated in response to exogenous WNT signalling (Fig. 6Ba-d′). We used the γ-secretase inhibitor LY411575 to inhibit Notch signalling (Curry et al., 2005) and found that the expression of both *Lfringe* (Notch target gene) (McGrew et al., 1998) and *cMyc* was lost in the treated explants (Fig. 6C). Finally, culture of CLE/cPSM explants with 100 nM RA for 6 h reduced *Fgf8* levels, as previously reported (Diez del Corral et al., 2003); however, it did not change *cMyc* levels, suggesting that within these progenitors it is unlikely that RA acts upstream of cMyc (Fig. 6D).

These experiments suggest that the three segmentation clock pathways (WNT, FGF and Notch) converge in regulating *cMyc* transcription in the NMPs and cPSM. Given the extensive crosstalk between these pathways (Akai et al., 2005; Bone et al., 2014; Katoh, 2007; Stulberg et al., 2012), each pathway could be acting directly or indirectly to regulate cMyc expression.

### Myc inhibition delays clock gene oscillations and slows somitogenesis

We then tested whether Myc plays a role in somitogenesis. We bisected E9.5 tails and incubated them for 4 h, with one half cultured in the presence of DMSO and the other in the presence of 10 μΜ JQ1 or 75 μΜ 10074G5. We then analysed the phase expression patterns of the Notch target clock gene, *Lfringe*, in the tail explants. Control explants formed at least one new somite pair during the 4 h incubation; however, Myc-inhibited explants displayed delayed *Lfringe* oscillations as compared with their control counterparts and, in some cases, also formed fewer somites (Fig. 6E).

We have previously published that regulation of Notch1 intracellular domain (NICD) turnover regulates the period of clock gene oscillations (Wiedermann et al., 2015). To see whether the observed delay in somite formation and clock gene oscillations upon Myc inhibition is linked to Notch signalling, we analysed levels of NICD in E9.5 tails cultured for 4 h in the presence or absence of Myc inhibitors by western blotting. Tail lysates incubated with either of the two small molecule inhibitors displayed higher levels of NICD, when compared with DMSO-treated tail lysates (Fig. 6F). To investigate whether the increased NICD levels result from new NICD production, we first incubated the explants with JQ1 or 10074G5 for 3 h and subsequently added the γ-secretase inhibitor LY411575 for 1 h. Control DMSO explants showed depletion of the NICD protein, whereas explants in which Myc activity was suppressed showed elevated NICD levels (Fig. 6F). Previously cMyc has been shown to repress *Notch1* transcription (Zinin et al., 2014). To test whether the increased NICD levels result from increases in *Notch1* or delta 1 (*Dll1*) transcription, we analysed *Notch1* and *Dll1* levels in CLE/cPSM explants treated for 6 h with 10 μΜ JQ1 or 75 μΜ 10075G5. We did not find significant changes in *Notch1* or *Dll1* transcript levels (Fig. 6G). These data suggest that the increases in NICD levels following Myc activity suppression are post-translational effects, and not a result of increased levels of *Notch1/Dll1* transcription.

### Conditional inducible cMyc depletion results in reduction of *Fgf8* expression levels

To study these diverse functions *in vivo*, we generated a conditional inducible mouse line to specifically genetically ablate cMyc expression in a spatially and temporally controlled manner from the tail of the postimplantation embryo. We employed an available transgenic *cMyc* mouse line, in which loxP sites have been placed on either side of exons 2 and 3 of the *cMyc* locus (Trumpp et al., 2001; hereafter referred to as the *cMyc^FL/FL^* allele). To specifically ablate cMyc from the embryonic tail, we used an available mouse line in which ERT2-CRE expression is conditionally under the control of the Nkx1-2 promoter (Rodrigo Albors et al., 2016 preprint). Nkx1-2 starts to be expressed in the CLE associated with the anterior primitive streak at E7, and continues to be highly transcribed here and at declining levels in the pre-neural tube domain at E8.5 (Rodrigo Albors et al., 2016 preprint). Tamoxifen-induced homologous recombination through CRE recombinase activity results in excision of the two exons and loss of function of the *cMyc* genetic product (Trumpp et al., 2001). Visualization of the CRE activation domain was possible through immunofluorescence labelling for the YFP protein; the *YFP* allele, controlled by the Rosa26 promoter, was activated for expression upon CRE-mediated excision of a loxP-flanked STOP sequence located upstream of the *YFP* locus (Rodrigo Albors et al., 2016 preprint; Srinivas et al., 2001).

Using ISH on E8.5 embryos, we verified a complete loss of *cMyc* transcript in the CLE of cMyc KO embryos, whereas gene expression was present in the somites and head (Fig. S4A). In addition, immunofluorescence labelling for GFP revealed intense staining in the CLE domain, with a few cells labelled in the neural tube, in accordance with Rodrigo Albors et al. (2016 preprint). The cMyc KO embryos at E8.5 were indistinguishable from control wild-type embryos, and loss of cMyc did not affect formation of the PSM, somites and neural tissue. These experiments suggest that acute cMyc loss from the E8.5 CLE does not affect short-term embryo development.

**Fig. 7.**
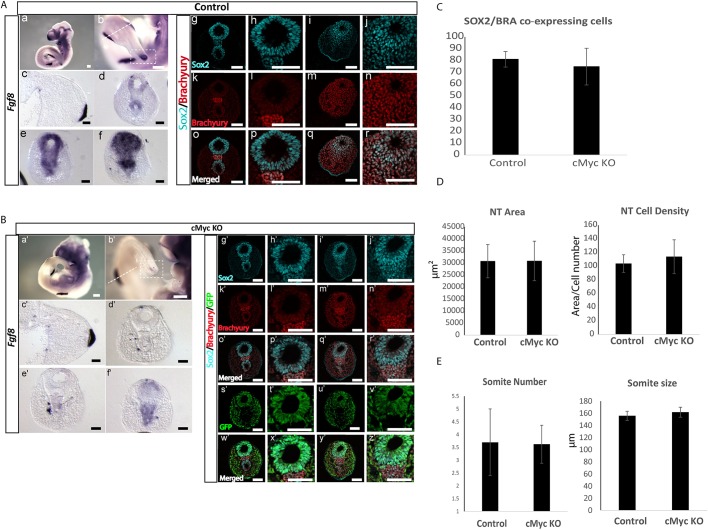
**Conditional inducible cMyc depletion from the CLE results in downregulation of *Fgf8* expression during axis elongation.** (A) (a-f) Representative images of E10.5 control embryos (*n*=3 embryos) labelled by ISH for *Fgf8* expression show high expression levels in the hind limbs and in the tail bud. (c) Transverse section in the limb bud region demarcated by the white dashed line in b. (d-f) Transverse sections of the CNH and tail bud demarcated by the white dashed line box in b. (g-r) Confocal images of transverse sections of the CNH and tail bud labelled by immunofluorescence for Sox2 and brachyury, showing a large number of co-expressing cells in the tail bud mesenchyme (data from ten confocal sections from two embryos). (B) Representative images of E10.5 cMyc conditional inducible mutant embryos (*n*=3 embryos) labelled by ISH for *Fgf8* expression. (a′-f′) High levels of *Fgf8* are detected in the hind limbs, whereas very low levels are detected in the tail bud. (c′) Transverse section in the limb bud region demarcated by the white dashed line box in b′. (d′-f′) Transverse sections of the CNH and tail bud demarcated by the white dashed line box in b′. (g′-z′) Confocal images of transverse sections of the CNH and tail bud labelled by immunofluorescence for Sox2, brachyury and GFP show large numbers of co-expressing cells in the tail bud mesenchyme. GFP cells are mostly absent from the CNH (s′), where *Fgf8* expression is still detected (f′) (data from ten confocal sections from two embryos). Scale bars: 100 μm. (C) Quantification of Sox2/brachyury co-expressing tail bud cells in control (*n*=2; eight sections) and cMyc KO (*n*=2; eight sections) embryos did not reveal differences. The counts are normalized to the total number of DAPI-stained nuclei. (D) Measurements of the neural tube (NT) area and cell density did not reveal differences between control (*n*=2; 25 sections) and cMyc KO embryos (*n*=2; 18 sections). (E) No differences between somite number (counted below hind limb level) or size were found between control (*n*=5) and cMyc KO embryos (*n*=8).

To investigate whether loss of cMyc from the E8.5 tail region affects embryogenesis during body axis elongation, we collected E9.5, E10.5 and E11.5 embryos. We found that cMyc KO embryos were of normal morphology, and formed all CLE derivatives (PSM, somites and neural tissue) (Fig. 7Aa,Ba′). In addition, the somite number and size, and the neural tube area and cell density, were not affected in embryos in which cMyc was conditionally ablated (Fig. 7).

Expression analysis of key genes that were affected in the explant experiments revealed a profound reduction of *Fgf8* PSM expression in E9.5 cMyc KO embryos (*n*=5 embryos) (Fig. S4B). In particular, expression was severely downregulated in the overlying caudal-most neuroepithelium, whereas the PSM was completely devoid of expression. Similarly, E10.5 cMyc KO embryos exhibited normal morphology, with severely reduced *Fgf8* PSM expression compared with that of control embryos (*n*=3/3 embryos) (Fig. 7Ab-e,Bb′-e′). *Fgf8* expression was spared only in the mesodermal compartment of the CNH (Fig. 7Bf′). *Fgf8* expression in the limb buds of E10.5 cMyc KO embryos was unaffected compared with that of controls (Fig. 7Aa-c,Ba′-c′).

To address whether cMyc depletion and subsequent *Fgf8* downregulation affected the presence or number of Sox2/brachyury co-expressing NMP cells, we carried out immunofluorescence labelling in E10.5 tails for Sox2, brachyury and GFP. GFP-expressing cells in the tail bud (indicative of cMyc depletion) still co-expressed Sox2 and brachyury, in a similar manner to wild-type embryos, suggesting that cMyc-mediated loss of *Fgf8* throughout almost all of the tail end of the embryo is insufficient to directly affect the NMP identity (Fig. 7Ag-r,Bg′-z′). Quantification of the Sox2/brachyury cells in tail bud sections of control and cMyc KO embryos did not reveal any differences (Fig. 7C). Interestingly, the mesenchymal domain that still expresses low levels of *Fgf8* in the cMyc KO embryos (Fig. 7Bf′) is almost devoid of GFP-expressing cells (Fig. 7Bs′), in accordance with the work of Rodrigo Albors et al. (2016 preprint), which showed that *Nkx1-2*-expressing cells do not contribute to this mesoderm compartment of the CNH. These FGF8-positive cells might serve, in part, to maintain the NMP progenitors in the KO embryos. To further decipher whether other NMP regulatory factors could compensate for *Fgf8* loss, we checked the expression of *Wnt3a* in KO embryos. Expression of this gene appeared unaffected (Fig. S4), suggesting that further compensatory mechanisms safeguard expression of this gene. It is possible that the prolonged persistence of the *Fgf8* mRNA (Dubrulle and Pourquié, 2004), which is still expressed at low levels in the cMyc KO embryos, could indirectly promote *Wnt3a* expression, as extensive crosstalk between WNT and FGF signals has previously been reported in these tissues (Aulehla and Pourquié, 2010; Gibb et al., 2010; Henrique et al., 2015).

## DISCUSSION

cMyc has been reported to be expressed in the early postimplantation epiblast (Clavería et al., 2013; Sancho et al., 2013) and to be a pluripotency factor in ESCs (Chappell and Dalton, 2013). The pluripotency factor Oct4 is still expressed in the CLE at E8.5, whereas its expression is downregulated at E9.5 (Aires et al., 2016). Thus, cMyc expression in the CLE coincides with the presence of other known pluripotency factors (Sox2, Oct4), further highlighting that the CLE shares characteristics of the early pluripotent epiblast (Gouti et al., 2015; Henrique et al., 2015). However, in contrast to Oct4 (Aires et al., 2016), we found that cMyc continues to be expressed in the tail bud during tail growth stages. We found that Myc functions to promote expression of factors that maintain the NMP pool (by sustaining *Wnt3a/8a* and *Fgf8* expression) and the NMP identity (Sox2/brachyury co-expression) by promoting *Sox2* expression. Interestingly, cMyc has been shown to positively regulate Sox2 expression (Lin et al., 2009) and to directly promote WNT signalling in mouse pluripotent stem cells (PSCs) (Fagnocchi et al., 2016). In the same context, cMyc is WNT regulated, and establishes a positive feedback WNT network that is indispensable for identity maintenance (Fagnocchi et al., 2016; Fagnocchi and Zippo, 2017). Similarly, our work shows that both WNT and FGF converge upstream of *cMyc* transcription suggesting that cMyc could be establishing and maintaining a Myc/WNT/FGF network operating in the NMPs. Given that secreted WNT/FGF act on the underlying mesoderm and control PSM maturation, it is possible that the aforementioned network operates in both of these adjacent progenitor domains (NMPs/cPSM), even though active transcription (of at least a subset of FGF components) is restricted to the overlying CLE (Dubrulle and Pourquié, 2004). In addition to WNT and FGF, we found that Notch activity is also important for cMyc transcription, in alignment with other published work identifying cMyc as a canonical Notch protein target, where again it operates in a positive feedforward loop (Palomero et al., 2006).

Very recently, two independent studies have highlighted a novel role for glycolytic metabolism during axis elongation (Bulusu et al., 2017; Oginuma et al., 2017). Inhibition of glycolysis resulted in loss of NMPs, premature differentiation towards the neural lineage and cessation of elongation (Oginuma et al., 2017). Myc is involved in the regulation of metabolism (Clavería et al., 2013; Dang et al., 2006; Eilers and Eisenman, 2008; Fagnocchi and Zippo, 2017; Hsieh et al., 2015), while recently, cMyc was found to link FGF signalling and glycolysis during vascular development in mice (Yu et al., 2017). In our study, we found that Myc activity is crucial for maintenance of mRNA levels of two key glycolytic genes (*Tpi1*, *Eno3*) that have been shown to exhibit graded rostrocaudal expression along the PSM (Oginuma et al., 2017). Taken together, these data suggest that, in the NMP and cPSM populations, Myc activity might regulate progenitor pool maintenance through the integration of proliferative WNT/FGF signals, as well as regulation of a specific subset of metabolic genes.

A striking finding was that Myc inhibition delayed dynamic clock gene expression across the PSM, which also coincided with increases in NICD levels. Interestingly, Myc activity suppression did not result in significant changes in *Notch1* mRNA levels, in contrast to a previous study indicating that cMyc is a negative regulator of *Notch1* expression in the chicken embryo neural tube (Zinin et al., 2014). A possible explanation for how Myc might affect NICD levels in the PSM could be through negative regulation of proteins mediating NICD turnover. We have previously shown that WNT or CDK protein inhibition (Gibb et al., 2009; Wiedermann et al., 2015) delays the periodicity of dynamic *Lfringe* clock gene expression across the PSM and that this phenotype is linked to inefficient NICD turnover. Given we demonstrate here that cMyc expression is WNT regulated in this tissue, and we find that Myc regulates transcription of the CDK inhibitor p21, it is possible to hypothesize that Myc acts downstream of WNT signalling and upstream of p21, which in turn controls downstream CDKs that regulate NICD phosphorylation and subsequent turnover. It would be interesting to investigate further whether Myc activity is crucial for proper timing of FGF and WNT ‘clock’ gene oscillations, which would provide insight into how the segmentation clock pathways are mechanistically linked.

Because cMyc is highly expressed in the CLE and persists in the tail bud (in contrast to MycN), we chose to acutely deplete cMyc from the embryonic tail region and study possible defects caused during the body axis elongation stages. Global cMyc KO embryos, are smaller than wild-type embryos and display multiple defects including abnormalities in neural tube closure (Davis et al., 1993). They do, however, survive to E10.5, possibly due to compensatory activity of MycN (Trumpp et al., 2001). However, Sox2-driven conditional cMyc depletion from the early mouse epiblast does not compromise embryo viability up to E11.5, and only confers defects in the hematopoietic lineage (Dubois et al., 2008). Here, we were able to highlight a direct requirement for cMyc, as a transcriptional regulator of *Fgf8* in the caudal neuroepithelium and tail bud in regions in which the Sox2/brachyury co-expressing NMPs reside. *Fgf8* expression was spared only in the mesodermal compartment of the CNH of KO embryos. Despite *Fgf8* loss throughout most of this caudal domain, the NMP pool was not compromised (at least at E10.5), which could be attributed either to compensation from the low level of FGF8 remaining, or other FGF factors that are present in the embryonic tail (Boulet and Capecchi, 2012; Naiche et al., 2011), or to uninterrupted WNT activity (for example through *Wnt3a*), which was not affected following genetic depletion of cMyc in this region and has been shown to be crucial for the NMP pool maintenance (Garriock et al., 2015). In addition, FGF8 has been previously shown to be dispensable for body axis elongation in the mouse (Perantoni et al., 2005), even though it is required for initial body axis specification and mesoderm migration through the primitive streak (Sun et al., 1999).

In summary, the current work uncovered multiple novel functional roles for Myc activity during body axis elongation. Future work should focus on deciphering the global Myc transcriptional signature within the progenitors that mediate this process.

## MATERIALS AND METHODS

### Mouse embryo collection and explant dissection

Pregnant female CD1 (between 10-20 weeks of age) mice were culled by cervical dislocation and the uteri were dissected and collected in phosphate buffered saline (PBS). Embryos of either 8.5, 9.5, 10.5 or 11.5 days postcoitum (dpc) were washed in Leibovitz's L15 medium (Life Technologies) or Dulbecco's modified Eagle medium (DMEM)-F12+0.1% Glutamax medium (Life Technologies), and collected in L15 or DMEM-F12 supplemented with 5-10% fetal bovine serum (FBS; Life Technologies. All animal procedures were approved by the Animal Scientific Procedures Act (1986, amended 2012).

For E8.5 CLE/cPSM explant dissection, mouse embryos were collected in L15 medium (Life Technologies). The embryos were first staged according to the somite number, and embryos at the five- to seven-somite stage were used. The tissue flanking the node and rostral to the tail bud mesoderm, which included the CLE and underlying mesoderm, was dissected and subsequently bisected along the longitudinal axis of the embryo to give right and left caudal embryo explant pairs. The explants were then individually embedded in freshly prepared rat tail-derived collagen mix [consisting of 71.5% rat tail collagen (Corning), 23.8% 5× L15, 23.8% acetic acid, 4.7% NaHCO_3_], and cultured for the appropriate timeframe at 37°C in a controlled atmosphere of 5% CO_2_ in air, in a culture medium consisting of Optimem (Life Technologies) supplemented with 5% FBS, 2 μΜ glutamine and 50 μΜ/ml gentamycin. For each pair, one explant was cultured in the appropriate volume of DMSO diluted in the above culture medium or in culture medium supplemented with the appropriate dilution of a small molecule inhibitor to suppress MYC [JQ1; a kind gift to K.G.S. from Dr James E. Brander (Harvard Medical School, MA, USA)] or 10074G5 (Sigma-Aldrich), WNT, Notch or FGF/ERK protein activity, as described in Table 1. For WNT signalling stimulation, the culture medium used was derived from L Cells (ATCC CRL-2648; hereafter referred to as control medium), or a DMEM-based WNT3A-enriched medium derived from L Wnt-3A cells (ATCC CRL-2647; hereafter referred to as WNT-conditioned medium) [supplemented with 10% FBS (Labtech), 2 mM L-glutamine (Lonza) and 1% penicillin/streptomycin (Lonza)] that had been prepared in house. For FGF stimulation experiments, recombinant h/m FGF8 (250 ng/ml; R&D Systems) was employed, as described in Diez del Corral et al. (2003).

**Table 1.**
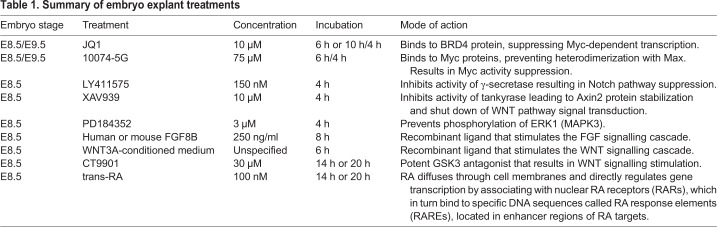
**Summary of embryo explant treatments**

At the end of the culture time, the embedded explants were fixed in 4% formaldehyde (Sigma-Aldrich) or 4% paraformaldehyde (Electron Microscopy) diluted in PBS, either for 2 h at room temperature or overnight at 4°C, if they were to be processed for ISH or for 2 h at 4°C if they were to be processed for immunofluorescence. They were directly lysed in RLT Buffer (Qiagen) if they were to be processed for RT-qPCR analysis. E9.5 half tail explants were micro-dissected and cultured as described in Bone et al. (2014).

### Differentiation of CLE/cPSM explants from E8.5 embryos towards mesoderm or neural lineages

CLE/cPSM explants derived from E8.5 embryos were cultured for 10 h at 37°C as described above. At the end of the 10 h incubation, both control (DMSO) and JQ1-treated collagen-embedded explants were rinsed in clean PSB and washed in PBS twice for 5 min. Subsequently, fresh culture medium [Optimem (Life Technologies), 5% FBS, 2 μΜ glutamine and 50 μΜ/ml gentamycin] supplemented either with 30 μΜ CT9901 (Tocris) or with 100 nM RA (Sigma-Aldrich) was added to the explants and they were further cultured for 14 h or 20 h at 37°C in a controlled atmosphere of 5% CO_2_ in air.

At the end of the culture time, the explants were fixed in 4% formaldehyde (Sigma-Aldrich) or 4% paraformaldehyde (Electron Microscopy) diluted in PBS, overnight at 4°C, and were processed for ISH.

### ISH

For ISH, the embryos and embryo-derived explants were first fixed overnight in 4% PFA at 4°C. ISH was carried out following standard procedures.

Colour revelation was performed after an overnight incubation with a 1:1000 dilution of the anti-digoxigenin antibody conjugated to alkaline phosphatase (Anti-Digoxigenin-AP, Fab fragments, Promega). Wild-type and genetically modified embryos were always treated in parallel and colour revelation was initiated and stopped simultaneously. Control and inhibitor-treated explants derived from each embryo were fixed simultaneously in 4% PFA and processed for ISH in parallel. Each explant pair was always treated simultaneously for colour revelation, which was stopped simultaneously for both explants. Colour revelation was stopped when adequate colour had developed in either the control or the treated explant of each pair. In the gain-of-function experiments for FGF and WNT stimulation, the treated explants were reproducibly saturated in each case, as they developed a lot faster than the control explants coloured in parallel. Explant expression data were double scored by two laboratory members independently.

### Immunocytochemistry

E8.5 whole mount, cryosections or explants of embryos were stained for immunofluorescence using primary antibodies against Sox2 [neural marker, raised in goat (Immune Systems, GT15098; LOT 909901)], brachyury [mesoderm marker, raised in rabbit (Santa Cruz Biotechnology, H-210; LOT H2514); or raised in goat (R&D Systems, AF2085; LOT KQP0319031)] and pH3 (Upstate Cell Signaling Solutions, 06-570; LOT32219). cMyc-positive cells were labelled using the monoclonal anti-c-Myc antibody [raised in rabbit (Abcam, Y69; LOT GR255057-5)]. Briefly, embryos and explants were blocked overnight in 10% heat-inactivated donkey serum (Sigma-Aldrich) diluted in PBT (1% Tween, 1% Triton, 2% bovine serum albumin diluted in PBS) at 4°C. All primary antibodies were used at a final concentration of 1:200 in blocking solution, and the embryos or explants were incubated overnight at 4°C. Following washes with PBST, the anti-donkey fluorescently conjugated secondary antibodies Alexa Fluor 488 and Alexa Fluor 594 (Invitrogen) were added at a final dilution of 1:200 in blocking solution and the tissues were incubated overnight at 4°C. Nuclei were counterstained using 4′,6-diamidino-2-phenylindole (DAPI) at a final dilution of 1:1000.

### TUNEL assay

The TUNEL assay was performed following a standard protocol optimized for tissue processing and using the ApopTag Kit (Millipore) according to the manufacturer’s instructions.

### Culture and differentiation of H9 hESCs (PSCs) to generate NMP-like cells

H9 (WA09) hESCs were purchased from Wicell and supplied at passage 24. The cells were thawed, transferred to DEF medium (Cellartis AB) and cell banks were prepared at passage 29. For routine production, the cells were used between passage 29 and 39. For quality control purposes, representative lots of the cell bank were thawed and tested for post-thaw viability, and to ensure sterility and absence of mycoplasma contamination. After two passages, the cell lines were tested for the expression of pluripotency markers (Oct4, Sox2, Nanog, SSEA-3, SSEA-4, TRA-1-60 and TRA-1-81) and differentiation markers [SSEA-1 (Fut4), HNF-3 beta (Foxa2), beta-III-tubulin and smooth muscle alpha-actinin] by immunofluorescence, and the ability to form all three germ layers when embryoid bodies are allowed to spontaneously differentiate in culture (immunofluorescence for HNF-3 beta, beta-III-tubulin and smooth muscle alpha-actinin).

H9 hESCs were maintained as feeder-free cultures in DEF-based medium (Cellartis DEF-CS) supplemented with 30 ng/ml bFGF (Peprotech) and noggin (10 ng/ml, Peprotech) on fibronectin-coated plates, and enzymatically passaged using TryPLselect (Thermo Fisher Scientific), and differentiated to the NMP-like state following the protocol from Verrier et al. (2018). All experiments with hESCs were approved by the UK Stem Cell Bank Steering Committee (SCSC14-28 and SCSC14-29).

### Image acquisition and analysis

Images of fluorescently labelled whole mounts, sections and explants of E8.5 embryos were taken using a Zeiss 710 confocal microscope equipped with a LASOS camera. Images of ISH were acquired using the Leitz DM RB Leica microscope, which is equipped with a Nikon D1X camera. Sox2/brachyury co-expressing cells and DAPI-positive nuclei were manually counted using ImageJ (https://imagej.nih.gov/ij/). Figure preparation was carried out using the free online software OMERO (www.openmicroscopy.org).

### RT-qPCR

Total RNA from H9 human PSCs and human NMPs or derived explants (6× CLE/cPSM explants per sample) was purified using an RNeasy Mini Kit (Qiagen) following the manufacturer’s instructions, and the concentration and quality of the eluted RNA was determined using the NANOdrop System. cDNA synthesis was performed using the Superscript III Kit (Invitrogen) in 0.25 μg, 0.5 μg or 1 μg of purified RNA according to the manufacturer’s instructions. RT-qPCR was performed using 1 μl of synthesized cDNA and 9 μl of Power SYBR Green PCR Master Mix (Life Technologies/Fisher), diluted in which were primers against the gene of interest (Table S1). A CFX96 thermocycler (Bio-Rad) was used for target cDNA amplification. Dilution curves for each primer set were performed to ensure that the primers were working at 100% efficiency, and the del-delcT method (Livak and Schmittgen, 2001) was used to analyse gene expression levels. Statistical significance was assessed either with the Student’s *t*-test or with analysis of variance (ANOVA).

### Western blotting of mouse E9.5 embryo tails

Protein extraction and western blotting was carried out following standard procedures using 10 μg of protein and the following dilutions of antibodies: 1:1000 of the rabbit anti-mouse NICD (Cell Signaling Technology) and 1:10,000 mouse β-actin (Proteintech).

### Generation of the inducible conditional cMyc mouse line

In order to genetically deplete cMyc from the tail end of the embryo, we utilized two available C56 mouse lines. One previously generated in the laboratory (Rodrigo Albors et al., 2016 preprint), in which CRE recombinase expression is driven under the control of the Nkx1-2 promoter (hereafter referred to as the *Nkx1-2^ERT2^^_CRE^* locus), which also carried a LoxP-flanked STOP sequence upstream of a EYFP reporter transgene of the Rosa26 ubiquitous promoter (hereafter referred to as the *YFP* locus) (Rodrigo Albors et al., 2016 preprint; Srinivas et al., 2001), and one in which LoxP sites have been placed on either side of exons 2 and 3 of the cMyc locus (hereafter referred to as the *cMyc FLOX^N^* locus) (Trumpp et al., 2001). Homozygote mice for the *cMyc FLOX^N^*, *Nkx1-2^ERT2_CRE^*, *YFP* alleles were morphologically normal and indistinguishable from C57 wild-type mice. Induction of homologous recombination was achieved by administering 200 μl of tamoxifen (Sigma-Aldrich), diluted in 10% ETOH/90% vegetable oil in a final concentration of 40 μg/ml, to the pregnant mother on 6 dpc and 7 dpc by oral gavage. Embryos were collected and analysed on 8.5, 9.5, 10.5 and 11.5 dpc. Validation of the region in which the *cMyc* locus was knocked out was achieved by immunofluorescence labelling against the YFP protein and by ISH against *cMyc*. Throughout the generation and maintenance of the mouse colony, ear biopsies were dissected from mouse pups for genotyping. DNA was extracted from the ear biopsies by direct addition of 20 μl of the MICROLYSIS mix (Cambio) in the biopsy sample. The PCR programs and primer sets for detection of the *cMyc FLOX^N^* locus are from Trumpp et al. (2001) and those for the *Nkx1-2^ERT2_CRE^* and *YFP* alleles are from Rodrigo Albors et al. (2016 preprint).

## Supplementary Material

Supplementary information
