# Supplementary material for: Myc activity is required for maintenance of the neuromesodermal progenitor signalling network and for segmentation clock gene oscillations in mouse
Source: Development. 2018 Jul 30;145(14):dev161091. doi: 10.1242/dev.161091 (PMC6078331; doi:10.1242/dev.161091)
Supplement: Supplementary information [file develop-145-161091-s1.pdf]

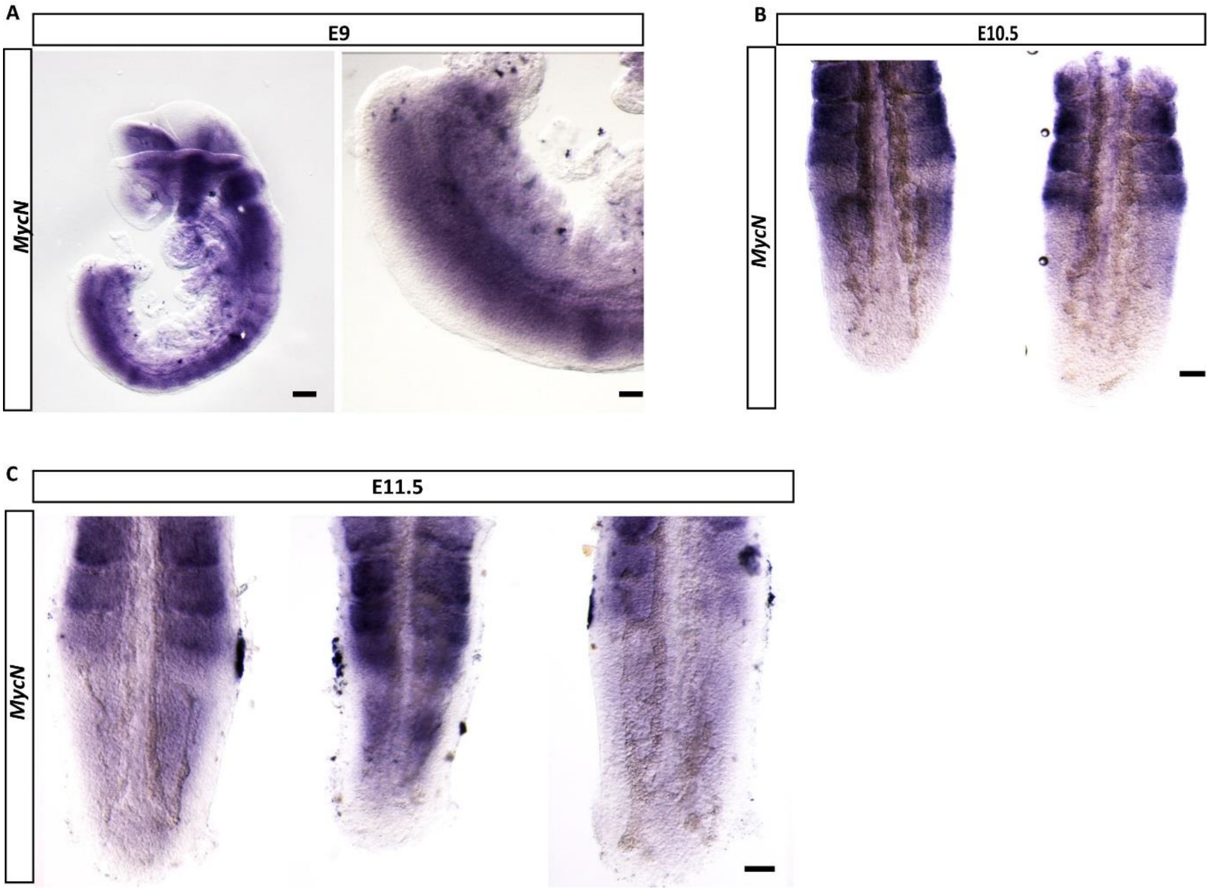

Figure S1. MycN expression at E9-E11.5

(A) Representative *in situ* hybridisation images for *MycN* at E9.5 (n=3) reveal an enrichment for *MycN* expression in the PSM compartment, and in particular a strong rostral domain of expression below the level of the last somite (white arrowhead), similarly to cMyc.
(B) Representative *in situ* hybridisation images of different embryo tails at E10.5 (n=5) *MycN* is highly expressed in the somites of the E10.5 embryo tails and in variable levels across the PSM. A strong band of mRNA expression was evident in some tails, below the level of the last somite (white arrowhead).
C) Representative *in situ* hybridisation images of different embryo tails at E11.5 (n=4) days of development. *MycN* is highly expressed in the somites of the E11.5 embryo tails and in variable levels across the PSM. A strong band of mRNA expression was evident in some tails, below the level of the last somite (white arrowhead).

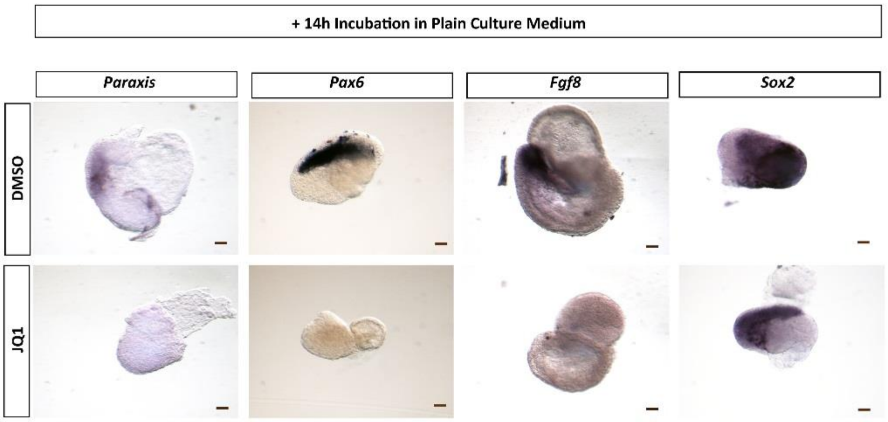

Figure S2. Wash out of JQ1 does not promote differentiation

Representative *in situ* hybridisation images of CLE/cPSM explants that were treated with (bottom row) 10 µM JQ1 or (top row) equivalent DMSO volume for 10h and subsequently cultured for further 14h in plain culture medium. In that timeframe explants that were initially incubated with JQ1 do not upregulate expression of *Paraxis* (n=0/2 embryos) or *Pax6* (n=0/3 embryos). Furthermore, they do not recover *Fgf8* expression (n=0/7 embryos) but they do express *Sox2* (n=4/4 embryos) at similar levels to their control counterparts. At this timeframe, JQ1 treated explants appear to be of a smaller size than the control DMSO explants.

Scale bars are 100 µm.

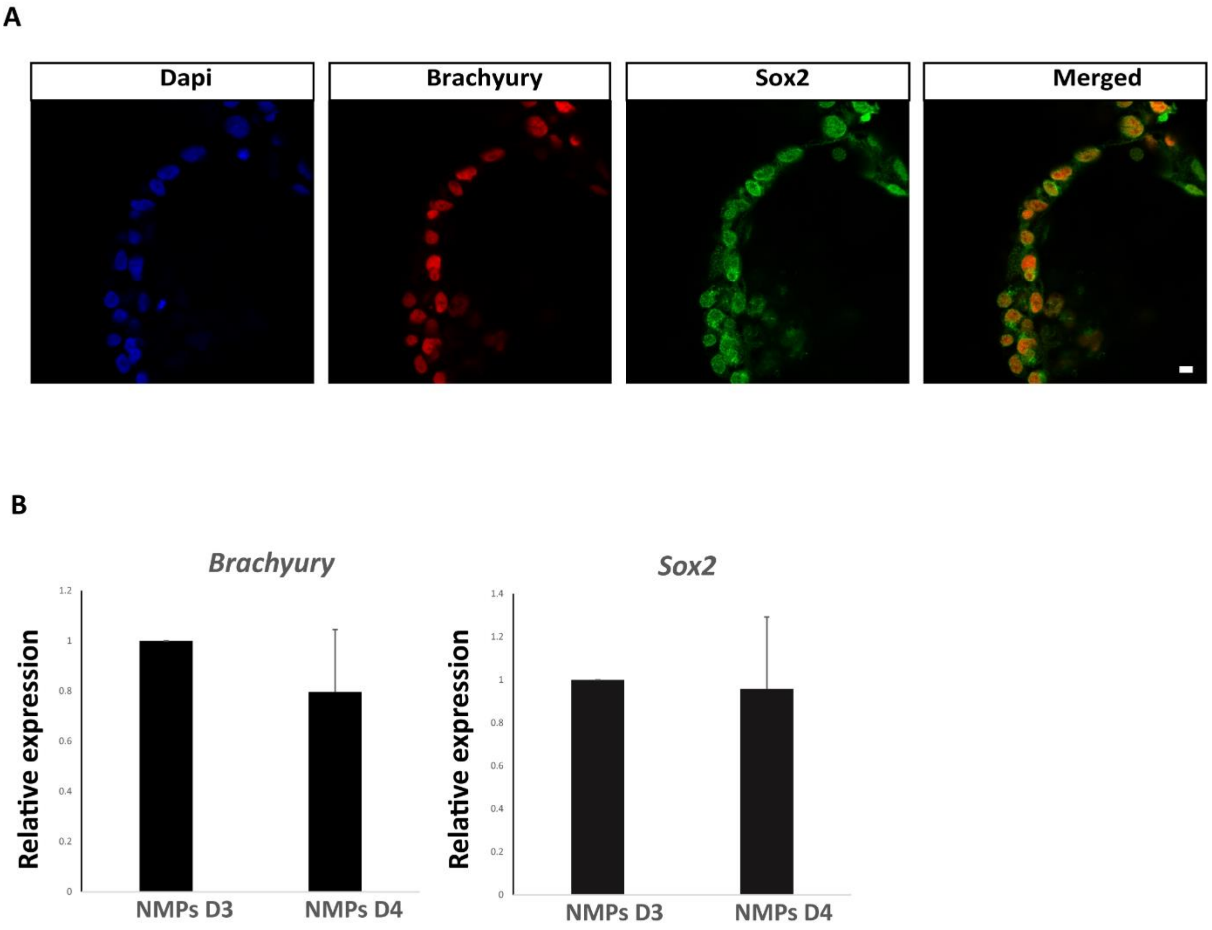

Figure S3. hNMP maintenance for 24h

(A) Representative confocal images of *in vitro* generated hNMPs from H9 pluripotent stem cells, show co-localisation of Sox2/Brachyury positive cells on Day3 of the differentiation protocol. Scale bar is 100 µm.

(B) qRTPCR expression analysis of Sox2 and Brachyury between Day3 and Day4 of the differentiation protocol shows that gene expression levels for these two genes remain unaltered in the 24h timeframe (data from two independent experiments, expression normalised to levels of PRT2 gene)

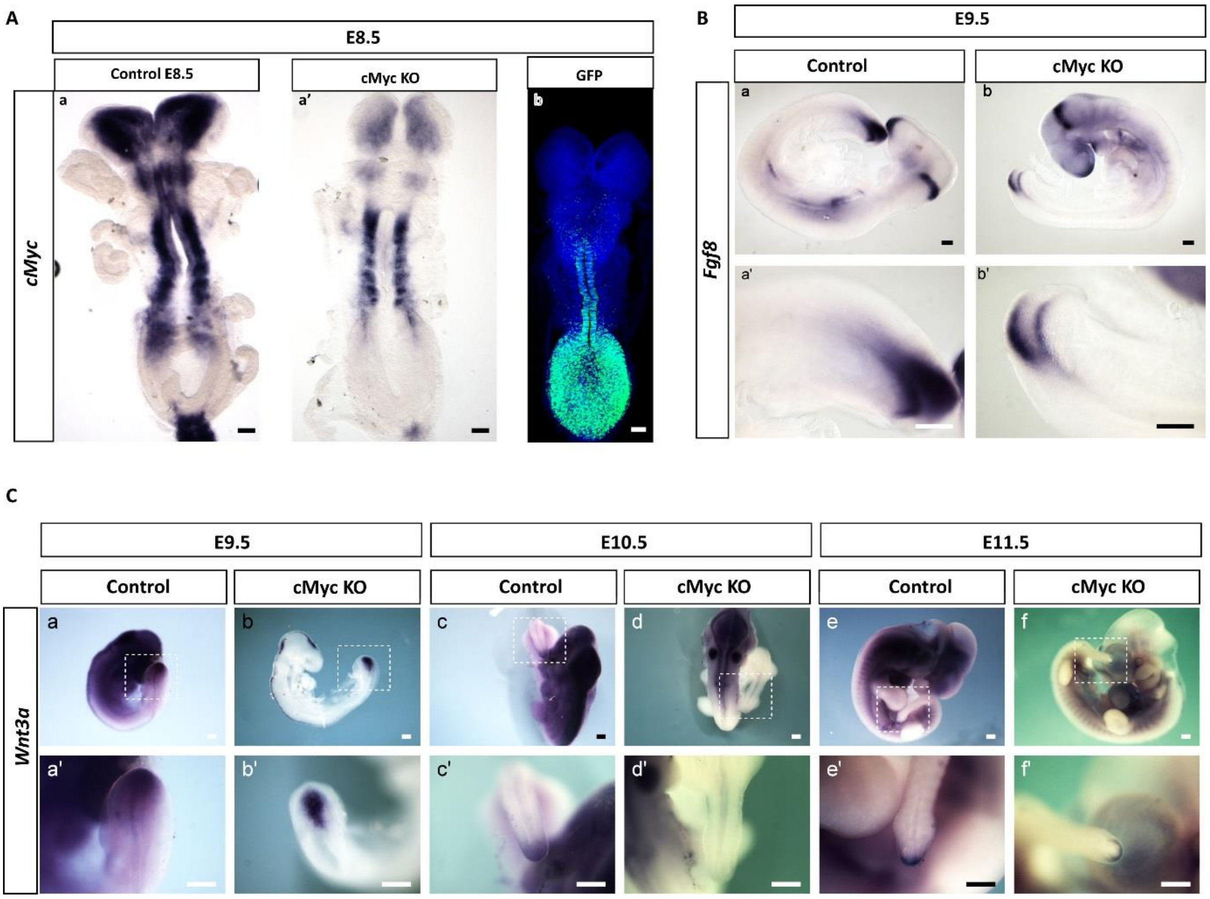

Figure S4. *Fgf8* but not *Wnt3a* is downregulated in conditional inducible cMyc KO embryos

A) Representative *in situ* hybridisation images of (a) Control Wild type (n=5) and (b) cMyc KO embryos (n=5) where a reduction of *cMyc* expression in the tail region is evident. Tamoxifen was administered at E6.5 and E7.5 days of development.

(b) Representative staining by immunofluorescence for GFP labels the CLE cells where recombination and subsequent cMyc depletion has taken place (n=5 embryos).

(B) Representative *in situ* hybridisation images for *Fgf8* expression in control (a,a') and cMyc KO embryos (b,b') at E9.5 show a reduction in *Fgf8* transcript levels specifically in the tail region (n=4 control and n=5 cMyc KO embryos)

(C) Representative images of control (a,c,e) and cMyc KO (b,d,f) E9.5, E10.5 and E11.5 embryos labelled by *in situ* hybridisation for *Wnt3a* expression (a'-f' panels are higher magnification images of the area denominated with the white dotted rectangle on a-f). We find very mild downregulation of *Wnt3a* expression in 1 out of 2 E9.5 embryos, 2/5 E10.5 and no downregulation in 3/3 E11.5 embryos. Scale bars are 100 µm.

Development • Supplementary information

Table S1. List of primers used

Gene	Forward sequence	Reverse sequence
**Human**		
brachyury	CCCGAAAGATGCAGTGACTT	CATCTCCACAGTTGGGTTCA
PRT2	TGACACTGGCAAAACAATGCA	GGTCCTTTTCACCAGCAAGCT
SOX2	TGGTACGGTAGGAGCTTTGC	GCAAGAAGCCTCTCCTTGAA
WNT8A	TGCAAGTTCCAGTTTGCTTG	CCATTGTTTGACCCATCACA
**Mouse**		
β-actin	CCACCAGTTCGCCATGGAT	GGCTTTGCACATGCCGGAG
brachyury	GCCAAAGAAAGAAACGACCAC	ATTGGGAATACCCCGGCTG
Sox2	CAGGAGTTGTCAAGGCAGAGA	CTTAAGCCTCGGGCTCCAAA
Oct3/4	GAGACTTTGCAGCCTGAGGG	CTTTCATGTCCTGGGACTCCTC
Cdx2	AGTCCCTAGGAAGCCAAGTGAAA	CAGCCGCTGATGGTCTGTGTA
cMyc	GTTGGAAACCCCGCAGACA	GGCTGTACGGAGTCGTAGTC
Wnt3a	GGAATGGTCTCTCGGGAGTT	CTTGAGGTGCATGTGACTGG
Pax6	CCATCACCAATCAGCATAGGAATC	AGGAGTTGCTGGTGAGAGTT
Wnt8a	TGGTGAACCTTCACAACAACA	ATGCTGCAGCTTCCTGAGAT
p21 (Cdkn1a)	CATTCCCTGCCTGGTTCCTT	CCTGTTCTAGGCTGTGACTGCTT
cyclin E1	CAGAGCAGCGAGCAGGAGA	CAGCTGCTTCCACACCACTG
Fgf8	TCATTGTGGAGACCGATACTTT	TTCTCCAGCACGATCTCTGT
Paraxis	GTGTAAGGACCGGAGGACAA	GATGGCTAGATGGGTCCTTG
Axin2	ACCTCAAGTGCAAACTCTCACCCA	AGCTGTTTCCGTGGATCTCACACT
Tpi1	TGAGCCGTTTCCACCGCCCTATTA	GCTCCAACCATGAGTTTCCAGCCC
Ldha	CACTGACTCCTGAGGAAGAGGCCC	AGCTCAGACGAGAAGGGTGTGGTC
Ldhb	ACAAGTGGGTATGGCATGTG	ACATCCACCAGGGCAAGTT
Slc2a1	CCAGCTGGGAATCGTCGTT	CAAGTCTGCATTGCCCATGAT
Eno1	TAGGCATCCACACCTGACCACCAG	GGGCTCCAGACACTAGCTGGGAAG
Eno3	TCAAGGCCAAGTATGGGAAG	TTCAGACGCAGCTACATCCA
Dll1	TCAGATAACCCTGACGGAGGC	AGGTAAGAGTTGCCGAGGTCC
